# A comprehensive assessment of exome capture methods for RNA sequencing of formalin-fixed and paraffin-embedded samples

**DOI:** 10.1186/s12864-023-09886-1

**Published:** 2023-12-15

**Authors:** Liang Zong, Yabing Zhu, Yuan Jiang, Ying Xia, Qun Liu, Sanjie Jiang

**Affiliations:** 1 Wuhan BGI Technology Service Co., Ltd. BGI-Wuhan, Wuhan, China; 2 College of Life and Health Sciences, Wuhan University of Science and Technology, Wuhan, China; 3 BGI Tech Solutions Co., Ltd. BGI-Shenzhen, Shenzhen, China

**Keywords:** FFPE samples, RNA sequencing (RNA-Seq), Gene fusion

## Abstract

RNA-Seq analysis of Formalin-Fixed and Paraffin-Embedded (FFPE) samples has emerged as a highly effective approach and is increasingly being used in clinical research and drug development. However, the processing and storage of FFPE samples are known to cause extensive degradation of RNAs, which limits the discovery of gene expression or gene fusion-based biomarkers using RNA sequencing, particularly methods reliant on Poly(A) enrichment. Recently, researchers have developed an exome targeted RNA-Seq methodology that utilizes biotinylated oligonucleotide probes to enrich RNA transcripts of interest, which could overcome these limitations. Nevertheless, the standardization of this experimental framework, including probe designs, sample multiplexing, sequencing read length, and bioinformatic pipelines, remains an essential requirement. In this study, we conducted a comprehensive comparison of three main commercially available exome capture kits and evaluated key experimental parameters, to provide the overview of the advantages and limitations associated with the selection of library preparation protocols and sequencing platforms. The results provide valuable insights into the best practices for obtaining high-quality data from FFPE samples.

**Supplementary Information:**

The online version contains supplementary material available at 10.1186/s12864-023-09886-1.

## Introduction

RNA sequencing has been developed as one of the most sensitive tools for gene expression analysis. Among the library preparation methods, the standard Poly(A) enrichment protocol provides a comprehensive and accurate view of polyadenylated RNAs. This method allows for simultaneous quantification of a multitude of RNA transcripts, enabling unbiased annotation of splicing variants, novel transcripts, and non-coding RNAs. It’s widely used in the investigation of human diseases, as well as the identification of novel drug targets and biomarkers. However, when working with human tissue specimens from biobanks, hospitals, and other clinical research facilities, the quality and yield of RNA can often be compromised due to several factors, including sampling techniques and preservation conditions, affecting the downstream bioinformatic analysis [1].

Fusion genes play a crucial role in tumorigenesis and are involved in approximately 20% of human cancer cases. The rapid and accurate identification of fusion genes holds significant promise for understanding cancer pathogenesis, enabling precise therapeutic interventions, and targeting drugs that can effectively inhibit these abnormal gene fusions [2]. There is great potential to fully assess the performance characteristics, including accuracy, reproducibility, and analytical sensitivity, of RNA-Seq for detecting the fusion events.

**Fig. 1:**
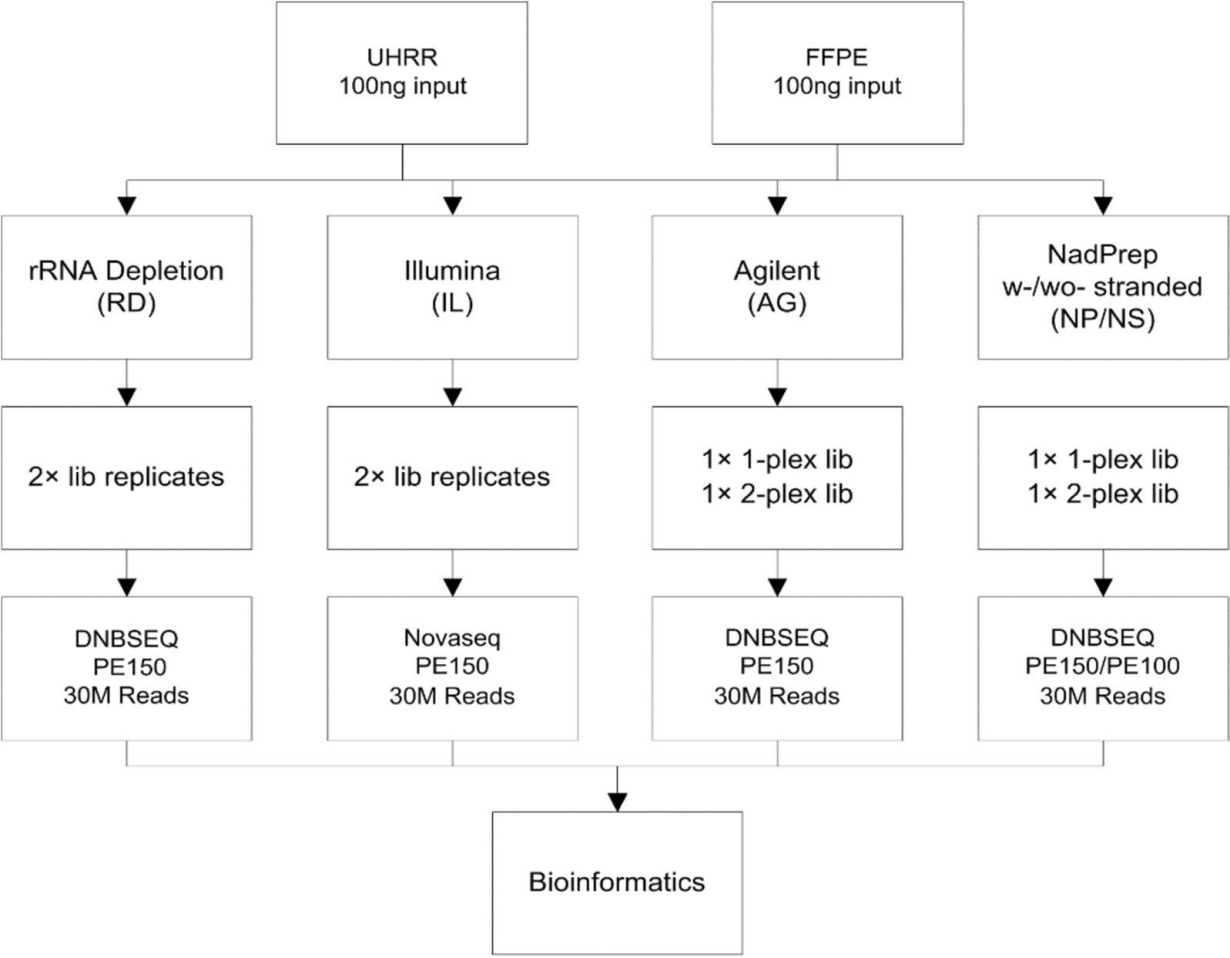
Experimental design of this study. 100 ng of heat-degraded Universal Human Reference RNA (UHRR) and one commercially available Formalin-Fixed Paraffin-Embedded (FFPE) sample were utilized as input materials. Three commercially available exome capture kits, namely Illumina (IL), Agilent (AG), and NadPrep (NP/NS), were employed. The rRNA depletion method (RD) was employed as the benchmark for bioinformatics analysis

In recent years, RNA-Seq library preparation protocols specifically designed for FFPE samples have been developed [3], such as the RiboZero and TruSeq RNA Exome kit (Illumina). RNA capture is a novel approach used to profile RNA samples of low integrity [4]. This method employs capture probes that target known exons, allowing for the enrichment of coding RNAs. Biotech brands, including Illumina, Agilent, and Nanodigmbio have developed commercial products, each utilizing distinct mechanisms and technologies. These products offer standardized, reproducible, and user-friendly protocols, making them suitable for gene expression studies conducted in various research settings. However, there is a lack of studies that investigate the differences of their applications and provide insights into fundamental technical questions [5]. To address this gap, we designed the study to evaluate the performance of three exome capture-based library preparation kits on human reference RNA from the Sequencing Quality Control consortium [6] and commercially available FFPE sections (Fig. 1). To our knowledge, this research is the first to compare the exome capture-based kits with the well-established rRNA depletion protocols specifically on FFPE samples. Additionally, we investigated the gene expression measurement in comparison to the TaqMan standard data and assessed the detection of fusion genes engineered in the FFPE reference RNA. The results aim to provide the scientific community with a comprehensive assessment of exome capture methods for RNA sequencing of FFPE samples.

## Methods

### Samples

To emulate the effects of formalin fixation-induced degradation, the human reference RNA (UHRR, #740000, Agilent Technologies) was incubated at 94 °C for 60 minutes to obtain fragmented RNA [7]. The peak observed in the Bioanalyzer (Agilent Technologies) trace of the fragmented sample was below 200 nt, and the peaks corresponding to 18S and 28S rRNA were absent.

The Onco Fusion FFPE RNA Reference Standard (#GW-OPSM001, GeneWell Biotechnology) containing multiple engineered clinically relevant fusion genes (Table 1) was procured, and total RNA was extracted using the RNeasy FFPE Kit (#73504, Qiagen) following the manufacturer’s instructions. The RNA Integrity Number (RIN) of the sample was 2.2, indicating significant degradation during the FFPE sections preparation [8].

**Table 1:** The list of fusion genes validated in the FFPE sample

Fusion genes	COSMIC ID (GRCh38·COSMICv91)	Experimental validated
EML4-ALK Fusion	COSF408	Yes
CCDC6-RET Fusion	COSF1271	Yes
SLC34A2-ROS1 Fusion	COSF1196	Yes
TPM3–NTRK1 Fusion	COSF1329	Yes
ETV6-NTRK3 Fusion	COSF571	Yes
CD74-ROS1 Fusion	COSF1200	Yes

### Library preparation

rRNA depleted RNA was generated with the Hieff NGS MaxUp Human rRNA Depletion Kit (#12257ES96, Yeasen Biotechnology), then strand-specific libraries were prepared using the Hieff NGS Ultima Dual-mode RNA Library Prep Kit (#12310ES96, Yeasen Biotechnology). For Illumina libraries, the TruSeq RNA Library Prep and Enrichment kit (#20020189, Illumina) was employed. Agilent and NadPrep libraries were generated using the Hieff NGS Ultima Dual-mode RNA Library Prep Kit, followed by the exome capture procedure using either SureSelect Human All Exon V6 (#5190–8864, Agilent Technologies) or Exome Plus Panel v2.0 (#1001841, Nanodigmbio). Multiplexing is a term used to describe the experimental strategy of pooling individual libraries together before probe hybridization. This approach is employed in Agilent and NadPrep kits to reduce the cost of the capture experiment, in which 2-plex means to pool two DNA libraries into one single tube. Illumina kits need to increase the consumption of the probes and hybridization reagent when pooling the libraries, multiplexing was not used in this study. The NadPrep libraries were divided into strand-specific and non-strand-specific groups. All protocols were conducted in accordance with the manufacturer’s instructions, with the recommended starting material of 100 ng of input RNA. The quality and yield of the prepared libraries were assessed using an Agilent 2100 Bioanalyzer.

### Sequencing

All the prepared DNBSEQ libraries were sequenced on the MGISEQ-2000 platform (MGI Tech) with PE150 cycles, except for the NadPrep libraries, which underwent an additional run with PE100 cycles. The Illumina libraries, on the other hand, were sequenced on the Novaseq 6000 platform (Illumina). Each library generated more than 30 million paired end reads.

### Data processing and quality control

Ribosomal RNAs were removed by bowtie2 (v2.4.5) alignment with the parameter “--very-sensitive-local --no-unal -I 1 -X 1000”. Duplication rate was calculated using fastp (v0.23.2). SOAPnuke (v1.5.6) was employed to filter out low quality reads based on the following conditions: 1) Reads containing over 50% of the length of 5′ or 3′ sequencing adapters; 2) Reads consisting of more than 1% of ambiguous bases; 3) Reads encompassing more than 20% of low-quality bases with quality score below 15. These filtering steps were carried out prior to any further analysis.

### Alignment and quantification

The high-quality reads obtained from each sample were aligned to the human reference genome (NCBI version GCF_000001405.39_GRCh38.p13) using HISAT2 (v2.2.1) with the parameter “--sensitive --no-discordant --no-mixed -I 1 -X 1000 --rna-strandness RF”. Sense rate and distribution across genome features were calculated by RSeQC (v4.0.0). Simultaneously, the reads were also mapped to the reference mRNA sequences using bowtie2 (v2.4.5) with the parameter: “--sensitive --dpad 0 --gbar 300 --mp 1,1 --np 1 --score-min L,0,-0.1 -I 1 -X 1000 --no-mixed --no-discordant -k 200”. RSEM (v1.3.1) was utilized to estimate the normalized gene abundances, represented as Fragments Per Kilobase Million (FPKM).

### Differential expressed gene (DEG) identification

The DEseq2 package (v1.31.16) was employed for the identification of DEGs. Two replicated libraries were considered as a single group for each sample. DEGs were determined based on the criteria of having a fold change greater than or equal to 1 and an adjusted *p*-value less than or equal to 0.05 when comparing the two groups.

### Fusion gene detection

Three distinct approaches were employed for the identification of fusion genes. The first one was EricScript (v0.5.5), which used the BWA aligner (v0.7.17) for mapping against the transcriptome reference. Samtools (v0.1.19) was utilized to handle the SAM/BAM files. BLAT (v35) was utilized for the recalibration of exon junction references. The second method was FusionCatcher (v1.33) which used two aligners (Bowtie v1.2.3 and STAR v2.7.2b) for read mapping and candidate fusion gene finding. FusionCatcher can filter out likely false positive candidate fusion genes of several conditions, including pseudogene, paralog gene or miRNA genes. The last method employed was STAR-Fusion (v1.12.0), which leveraged the output generated by the STAR aligner (v2.7.8a) to map both junction reads and spanning reads. These reads were mapped against a reference annotation set GRCh38_gencode_v37_CTAT_lib_Mar012021.plug-n-play from the Trinity Cancer Transcriptome Analysis Toolkit (CTAT) genome lib. All the methods were implemented using default parameter settings.

## Results

In order to evaluate the performance of exome capture-based RNA-Seq methods in profiling FFPE samples, we conducted a meticulous technical assessment of the three distinct protocols mentioned above, namely Illumina (IL), Agilent (AG), NadPrep with both stand-specific treatment (NP) and non-strand-specific treatment (NS) on human reference RNA (UHRR) and commercially available FFPE RNA (FFPE). The schematic of the workflow along with statistical information at each library preparation step are presented in Table S1. We utilized the dataset to evaluate and assess the performance of RNA-Seq library preparation protocols. First, we examined the protocols’ ability to maintain consistent alignment rates. Second, we assessed the accuracy of these protocols in calculating gene expression by comparing the results to TaqMan data. At last, we explored the protocols’ capability to identify fusion genes that have been experimentally validated within the samples.

### Alignment statistics

Overall, we prepared a total of 20 libraries for sequencing, both the DNBSEQ and Novaseq platforms generated comparable high-quality reads. The Novaseq libraries exhibited a significantly higher duplication rate compared to the DNBSEQ libraries (Table S1). This can be attributed to the exponential amplification during the clustering process [9], which may have an impact on the saturation of data, particularly when sequencing resources are limited. We initially examined the overall alignment rates to the human genome (Table 2). The results demonstrated that all the protocols exhibited good performance, indicated by the high alignment rates greater than 90%. The AG libraries exhibited relatively higher rRNA contamination, suggesting lower specificity of the exome capture procedure in this protocol.

**Table 2:** The alignment statistics to the human genome

No.	Library	Genome Mapping Rate (%)	Gene Mapping Rate (%)	Sense Rate (%)	Genes detected number	Transcripts detected number
1	FFPE_RD_1	98.07	32.56	96.27	16,077	53,547
2	UHRR_RD_1	97.64	40.52	96.26	17,157	59,524
3	FFPE_RD_2	97.57	33.46	95.87	16,108	53,972
4	UHRR_RD_2	97.77	40.53	96.24	17,158	60,039
5	FFPE_IL_1	95.52	79.47	99.51	15,467	47,185
6	UHRR_IL_1	92.12	76.89	99.39	16,420	48,113
7	FFPE_IL_2	95.03	79.13	99.52	15,530	47,692
8	UHRR_IL_2	94.24	77.03	99.42	16,374	47,190
9	FFPE_AG_1	96.01	73.71	97.38	16,683	55,486
10	UHRR_AG_1	97.56	75.76	97.54	17,196	59,114
11	FFPE_AG_2	97.15	73.7	96.71	16,655	53,422
12	UHRR_AG_2	96.8	73.83	97.37	17,038	56,474
13	FFPE_NS_1	92.71	74.12	64.12	17,410	59,360
14	UHRR_NS_1	91.03	70.31	68.08	17,799	60,125
15	FFPE_NS_2	92.77	73.64	70.36	17,595	59,859
16	UHRR_NS_2	91.54	71.57	71.48	17,865	60,590
17	FFPE_NP_1	95.93	75.48	97.99	16,885	57,648
18	UHRR_NP_1	96.34	76.48	98.22	17,303	60,355
19	FFPE_NP_2	95.66	73.33	98.08	16,699	54,446
20	UHRR_NP_2	96.05	76	98.35	17,136	57,652

The sense rate is a metric that calculates the percentage of aligned forward reads mapped to the antisense gDNA strand and the percentage of aligned reverse reads mapped to the sense gDNA strand. Strand-specific RNA-Seq protocols offer the advantage of resolving read ambiguity in cases where overlapping genes are transcribed from the opposite strands. This specificity enhances the accuracy of gene quantification and prediction of fusion genes. The IL libraries showed the highest sense rate, indicating a more efficient strand-specific library preparation process [10]. Meanwhile, the IL libraries yielded the lowest count of identified genes and transcripts, potentially be attributed to the different design of targets and exome panels among these kits. We can make a preliminary inference about the genes for which Illumina kits lack probes, by noting a particular gene that exhibits consistent expression across all other libraries, yet in IL libraries the FPKM value is recorded as zero. Most of the selected genes are non-annotated, while some of them, such as gene *C4orf48* may be disease relevant and important for clinical studies [11]. The NP libraries exhibited a higher genome mapping rate, but lower number of genes detected than the NS libraries. These findings align with previous studies based on Poly(A) RNA sequencing [12].

In addition, the protocols showed notable distinctions in the proportions of reads aligned to exons, introns, and other intergenic regions (Table S2). For NS and NP libraries, the percentages of reads that aligned to exons were above 94%, indicating the highest efficiency of the exome pull down by the capture approach. As anticipated [13], with the RD libraries, we observed that approximately half of the reads mapped to exons, while the remaining reads predominantly mapped to intronic regions (ranging from 32 to 42%).

### Transcript coverage

**Fig. 2:**
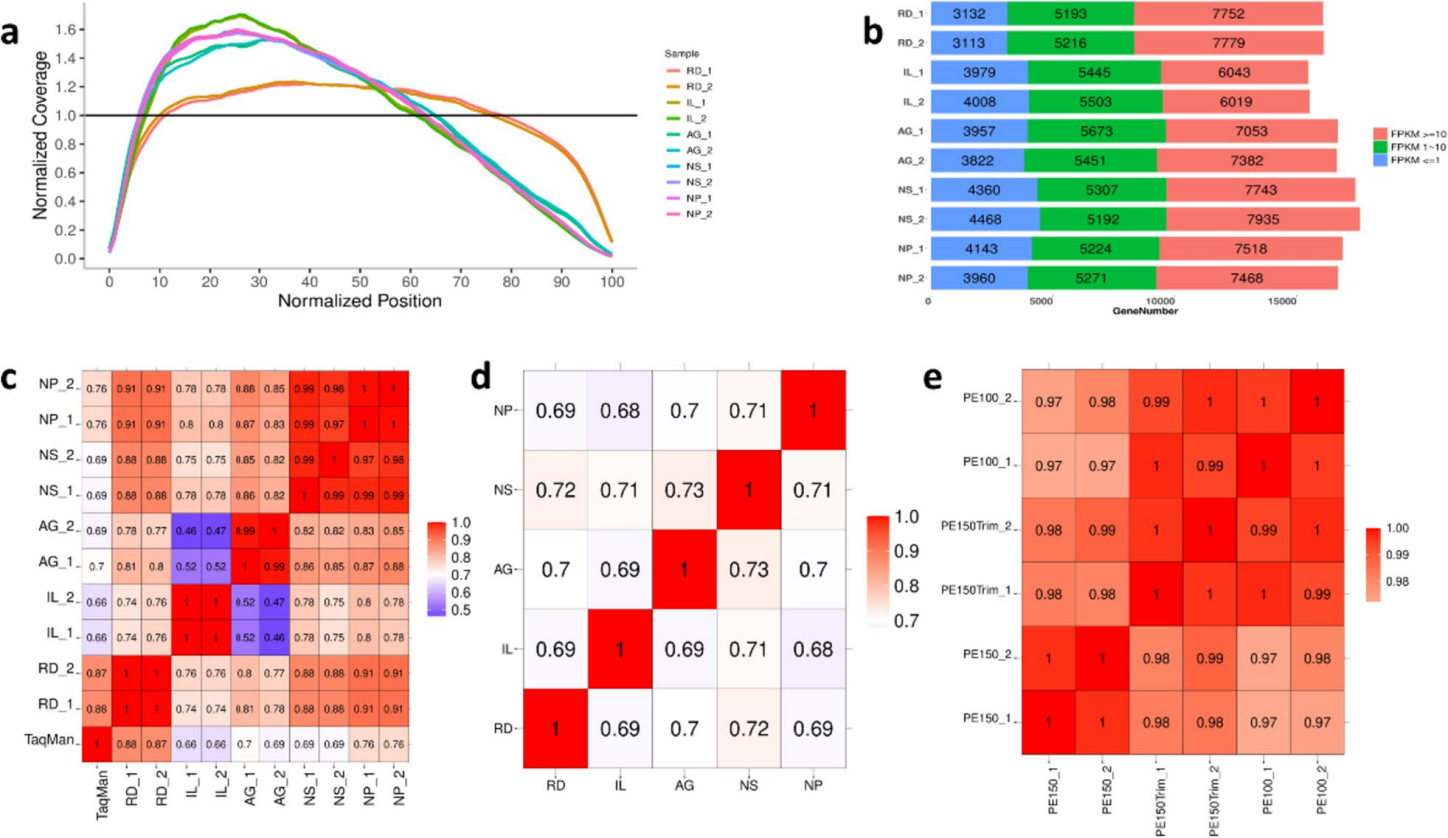
Statistics of alignment and expression of the experiments. **a** Normalized transcript coverage of UHRR libraries. **b** Expression level of genes detected in UHRR libraries. Correlation heatmap **c** between UHRR libraries and TaqMan data, **d** of fold-change values of genes between the two samples (FFPE vs UHRR), and **e** among NP libraries with different read lengths

We proceeded to assess the coverage across the full length of the genes and observed that all the protocols demonstrated broad and uniform transcript coverage (Fig. 2a). Notably, the exome capture-based protocols exhibited a slight 5′ bias, which can be attributed to the second structure of the transcripts and the mechanism of reverse transcription [14]. In other words, a higher proportion of reads mapped to the 5′ region of the transcripts compared to other methods, particularly those utilizing Poly(A) enrichment procedures. It is well established that with Poly(A) selected mRNA, the sensitivity of fusion detection is determined by the level of coverage at the position where the gene fusion resides, and this coverage decreases with the distance from the 3′ end of the mRNA, when the input RNA is in degradation [15]. Furthermore, the proportion of reads mapping to coding regions was significantly elevated comparing to RD libraries, making the exome capture-based protocols ideal for gene fusion detection in FFPE derived or other highly degraded samples.

### Gene expression

We first investigated the abundance distribution of all the genes detected by different protocols [16] and categorized them into groups based on their respective expression levels (Fig. 2b). Notably, the IL libraries exhibited the largest proportion of genes with low FPKM values (less than 1), when detecting the smallest total number of genes. This observation highlights a potential concern since clinical studies often establish thresholds for gene expression levels, like FPKM greater than 0.3 or 0.5, due to the methodology’s high sensitivity and inherent false-positive rate [17]. The mechanical filtering process may lead to the exclusion of clinical-relevant targets with low abundance or insufficient full-length coverage. This may further compromise the ability of IL libraries to identify rare fusion events.

We then assessed the agreement between the protocols by calculating the Pearson coefficient with all the UHRR libraries and the TaqMan reference data [18]. The exome capture-based libraries exhibited lower correlation (ranging from 0.66 to 0.76) than the RD libraries (exceeding 0.87). When comparing the different protocols against each other, the gene expression values exhibited significant protocol-specific biases, leading to a reduced agreement (correlations ranging from 0.46 to 0.88). However, the consistency within replicates and multiplexing was ideal, with correlations greater than 0.99 (Fig. 2c).

Finally, we assessed the agreement between the protocols by calculating the Pearson coefficient of the fold-change values of the differentially expressed genes between the two samples (FFPE vs UHRR, Fig. 2d)**.** The results demonstrated that the fold-change values correlated acceptably across the entire dynamic range of expression between the protocols (correlations ranging from 0.68 to 0.73), suggesting the possibility of comparing the DEGs of paired samples using different methods when the consistency of the whole study cannot be guaranteed.

### Fusion gene detection

**Fig. 3:**
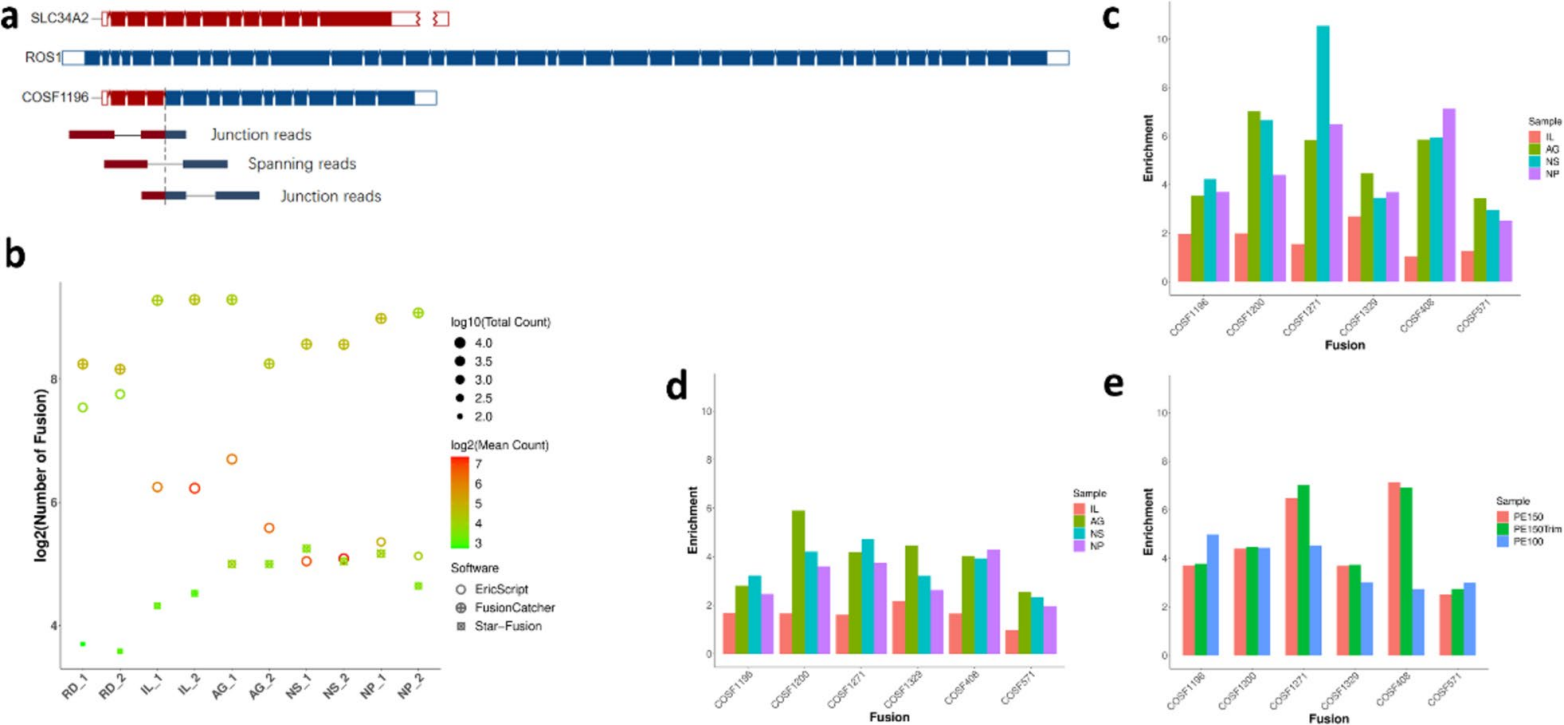
Statistics of fusion genes detection. **a** Diagram of COSF1196 transcript, depicting spanning and junction reads used to identify fusion genes. **b** Statistics of fusion events and the corresponding supporting read counts of all FFPE libraries by different pipelines. The circular diameter represents the total read counts for the cumulative fusion events, while the color corresponds to the mean read counts supporting each individual fusion event. Enrichment of 6 fusions between exome capture-based and rRNA depleted libraries calculated by **c** Star-Fusion, **d** FusionCatcher, and **e** using reads of different lengths

Multiple bioinformatic pipelines have been developed to identify candidate fusion genes from RNA-Seq data [19]. Predicted fusions is typically supported by fragments found as junction reads that directly overlap the splicing site, or as spanning reads where each pair of reads maps to the opposite partner of the fusion genes (Fig. 3a). In this study, we assessed three most cited methods for fusion detection: EricScript [20], FusionCatcher, and Star-Fusion. EricScript and FusionCatcher called more fusion genes than Star-Fusion (Fig. 3b), but for FFPE samples, EricScript reported only 1 of the 6 validated transcripts, while FusionCatcher and Star-Fusion reported all the 6 events (Table S3). Although it could be argued that these fusions represent a limited target reference for method comparison, EricScript exhibited lower sensitivity and higher false-positive rate. Notably, EricScript also reported fusion transcripts involving opposite partners of HLA-C and HLA-A with EricScore greater than 0.95, indicating a poor performance in distinguishing highly homologous genes. On the other hand, FusionCatcher reported more than 300 fusion candidates per library, although the majority of which were remarked as exonic or in-frame, researchers would face significant challenges in filtering and validating all these predictions.

We then examined the target enrichment rate of different protocols by comparing the Fusion Fragments Per Million mapped reads (FFPM) from exome capture-based and rRNA depleted data (Table S3 and Fig. 3c, d), finding that Star-Fusion exhibited better reproducibility in replicate or multiplexed libraries, and achieved a mean 1.75, 5.03, 5.63 and 4.65-fold enrichment for the IL, AG, NS and NP libraries respectively, illustrating the advantage of exome capture-based protocols in fusion genes detection compared to the conventional methods, particularly for highly degraded samples [21].

### Impact of read length

The length of the sequencing reads is a crucial factor to consider when conducting RNA-Seq experiments. Previous studies suggest that for generating a list of DEGs, 50 bp single-end reads are generally sufficient [22]. However, for isoform detection, longer reads are preferred to capture comprehensive information, while longer reads are not necessarily significantly better than shorter reads for differential expression analysis.

The DNBSEQ series of sequencers, including DNBSEQ-T7, MGISEQ-2000, and DNBSEQ-G99, offer a range of sequencing throughputs and read lengths for various research applications. In clinical occasions, researchers often face the decision between PE100 and PE150 run cycles, considering factors like sample quality, experimental costs, turnaround time requirements, and the challenges of pooling adequate libraries to fulfill a sequencing run. To investigate the impact of read length on FFPE RNA-Seq outcomes, we performed an additional MGISEQ-2000 PE100 run for NP libraries, made up three groups of NP data with different read lengths: PE150, PE150 trimmed into PE100 (PE150Trim) and PE100. The overall statistics of the sequencing results are presented in Table S4.

As expected, the PE100 data exhibited improved performance in terms of adapter contamination, reference alignment and number of genes detected. Nevertheless, it did not show significant changes in the accuracy of expression measurement (Fig. 2e) and the target enrichment rate of fusion genes (Table S3 and Fig. 3e). These findings suggest that while PE100 data improvements are beneficial for certain aspects, they may not provide additional advantages for clinical study applications. At the meantime, when compare FFPE_PE150_1 vs FFPE_PE100_1 (Table S5), there were 8904 specifically detected transcripts in PE150 data compared to PE100 data, hundreds of longer isoforms of clinically relevant transcripts can only be detected in PE150 data with low to median abundance (FPKM≥1), suggesting the potential of longer reads in disease studies.

## Conclusions

In this study, we present a comprehensive assessment that encompasses all crucial factors to be considered in RNA-Seq experiments to optimize the utilization of clinical FFPE samples. Researchers are provided with the opportunity to make informed decisions regarding the selection of capture probes, library preparation and multiplexing, sequencing parameters, and bioinformatic pipelines, especially when sample quality is severely compromised (Table 3). For fusion genes detection, prudent filtering techniques and critical experimental validations are imperative to ensure accuracy and reliability of the results [23]. We recommend the adoption of exome capture-based RNA-Seq protocols when the input sample are not suitable for conventional methods. Although the commercial kits offer advantages in the sequencing depth of coding regions, the considerations of inconsistent capture efficiency and rRNA residue merit careful attention and pre-experiment contemplation to ensure the optimal application of the selected protocols. We look forward that exome capture-based RNA-Seq methodologies will experience growing adoption in clinical settings for the diagnosis of fusion genes, further advancing our understanding of fusion gene biology, and enhancing cancer diagnostics.

**Table 3:** Summary of the decisions in exome capture-based protocols

Protocols	Parameters	Pros	Cons
Exome Panels	Illumina	Highest sense rate	Lowest target enrichment
	Agilent	Most cited panel design	Highest rRNA contamination
	NardPrep	Highest capture efficiency	None
Library Preparation	Strand-Specific	Increased genome mapping rate	Reduced transcripts identification
	Multiplexing	Comparable and low-cost libraries	Pilot study needed before experiments
Sequencing	Illumina	High quality reads	High duplication rate
	DNBSEQ	Flexibility of throughputs and read lengths	None
	Shorter Reads	Increased reference alignment	Elevated sequencing cost

### Supplementary Information

**Additional file 1.**

## Data Availability

All sequencing data have been deposited in the CNGB Nucleotide Sequence Archive (https://db.cngb.org/cnsa) under the accession number CNP0004621.
